# Stop smoking practitioner consensus on barriers and facilitators to smoking cessation in pregnancy and how to address these: A modified Delphi survey

**DOI:** 10.1016/j.abrep.2019.100164

**Published:** 2019-01-29

**Authors:** Libby Fergie, Katarzyna A. Campbell, Tom Coleman-Haynes, Michael Ussher, Sue Cooper, Tim Coleman

**Affiliations:** a Division of Primary Care, School of Medicine, University of Nottingham, UK; b Population Health Research Institute, St George's University of London, UK; c Institute for Social Marketing, University of Stirling, UK

**Keywords:** Smoking cessation, Pregnancy, Modified Delphi, practitioners' consensus, Barriers/facilitators, Behaviour change techniques

## Abstract

**Introduction:**

Pregnant women can experience barriers and facilitators towards achieving smoking cessation. We sought consensus from smoking cessation practitioners on how influential pre-identified barriers and facilitators can be on pregnant women's smoking behaviour, and how difficult these might be to manage. Suggestions for techniques that could help overcome the barriers or enhance the facilitators were elicited and consensus sought on the appropriateness for their use in practice.

**Methods:**

Forty-four practitioners who provided cessation support to pregnant women completed a three-round modified Delphi survey. Round one sought consensus on the ‘influence’ and ‘difficulty’ of the barriers and facilitators, and gathered respondents' suggestions on ways to address these. Rounds two and three sought further consensus on the barriers and facilitators and on ‘appropriateness’ of the respondent-suggested techniques. The techniques were coded for behaviour change techniques (BCTs) content using existing taxonomies.

**Results:**

Barriers and facilitators considered to be the most important mainly related to the influence of significant others and the women's motivation & self-efficacy. Having a supportive partner was considered the most influential, whereas lack of support from partner was the only barrier that reached consensus as being difficult to manage. Barriers relating to social norms were also considered influential, however these received poor coverage of respondent-suggested techniques. Those considered the easiest to address mainly related to aspects of cessation support, including misconceptions surrounding the use of nicotine replacement therapy (NRT). Barriers and facilitators relating to the women's motivation & self-efficacy, such as the want to protect the baby, were also considered as being particularly easy to address. Fifty of the 54 respondent-suggested techniques reached consensus as being appropriate. Those considered the most appropriate ranged from providing support early, giving correct information on NRT, highlighting risks and benefits and reinforcing motivating beliefs. Thirty-three BCTs were identified from the respondent-suggested techniques. ‘Social support (unspecified)’, ‘Tailor interactions appropriately’ and ‘Problem solving’ were the most frequently coded BCTs.

**Conclusions:**

Involving partners in quit attempts was advocated. Existing support could be potentially improved by establishing appropriate ways to address barriers relating to pregnant smokers' ‘social norms’. In general, providing consistent and motivating support seemed favourable.

## Highlights

• Significant others, women's motivation and self-efficacy influence quit attempts.
• Having an unsupportive partner was considered the most difficult barrier to address.
• Suggestions on how to address barriers relating to social norms were lacking.
• Giving consistent support and boosting women's motivation to quit were advocated.
• Enhancing women's knowledge on NRT use was deemed easy to do and beneficial.

## 1 Introduction

Currently 13% of women in England smoke (NHS Digital, 2018a), around 10.5% continue to smoke during pregnancy (NHS Digital, 2018b). Smoking during pregnancy has serious health implications for both the mothers and babies (NHS Digital, 2018b). The mother's risks of pregnancy related complications including placenta abruption, placenta previa, miscarriage, stillbirth, ectopic pregnancy, and premature labour, are increased (Cnattingius, 2004; World Health Organization, 2013). For the infants, the risks of low birth weight, birth defects such as cleft lip and palate (Maretelli et al., 2015), asthma (World Health Organization, 2013), cognitive impairments (Knopik et al., 2016), developing childhood malignancies (Chu, Wang, Han, et al., 2016; Jauniaux & Burton, 2007) and becoming a smoker later in life are increased (Leonardi-Bee, Jere, & Britton, 2011). Thus, smoking during pregnancy is a considerable health concern (World Health Organization, 2013), and in response, Public Health England aims to reduce the prevalence to 6% or below by 2022 (Department of Health England, 2017).

Although the overall prevalence rates in the country have dropped from 15.8% in 2006/07 (NHS Digital, 2018b), only 31 of 195 areas in England have, to date, met the 6% or below target (NHS Digital, 2018b). Areas to have achieved this target are mainly in Southern parts of the country (NHS Digital, 2018b) where levels of deprivation are relatively low (Department for Communities and Local Government, 2015), whereas areas that have not are mainly around Northern England and the Midlands (NHS Digital, 2018b) where levels of deprivation and prevalence of smoking are relatively high (Department for Communities and Local Government, 2015). Examples of this include London and the surrounding areas having prevalence rates of between 2.3% and 2.7%, whereas districts in and around Blackpool and North Nottinghamshire have prevalence rates of between 23.2% and 24.9% (NHS Digital, 2018b).

Although pregnancy is normally a highly motivating time to quit (Solomon & Quinn, 2004), women can experience barriers and facilitators which may make cessation attempts more difficult or easier at this time (Flemming, McCaughan, Angus, & Graham, 2015). Findings from a qualitative review looking at barriers and facilitators from pregnant smoker's perspectives (Flemming et al., 2015) and a UK expert group meeting study (Campbell et al., 2018), identified 34 barriers and facilitators (23 barriers, 11 facilitators) specific to achieving smoking cessation during pregnancy. These ranged from aspects within the women's network (e.g. ‘smoking is a social norm’) to parenting responsibilities (e.g. ‘desire to protect the baby from harm’) (the full list is given in Supplement 1).

Behavioural support interventions, consisting of behaviour change techniques (BCTs), can be effective in helping pregnant women overcome barriers and optimise facilitators to successfully achieve cessation (Chamberlain et al., 2017; Lorencatto, West, & Michie, 2012). A BCT is the smallest, active component which can be delivered in an intervention either by itself or in conjunction with other BCTs in order to achieve the intended behaviour change outcome (Michie et al., 2011). Some BCTs have been shown to be effective when used in smoking cessation interventions for pregnant women (e.g. facilitate action planning/identify relapse triggers) (Campbell et al., 2018; Lorencatto et al., 2012). However, a review of English Stop Smoking Services (SSS), found few of these BCTs were being used in practice (Lorencatto et al., 2012). Furthermore, there are inconsistencies in the support offered to pregnant women across English SSSs which could potentially be improved and standardised (Fahy, Cooper, Coleman, Naughton, & Bauld, 2014).

In order to increase the effectiveness of behaviour change interventions, it is important that any potential barriers and facilitators are taken into consideration (Craig et al., 2017; Michie, Atkins, & West, 2014). However, as assessing all potential barriers and facilitators can be a lengthy, cumbersome and not always an advantageous process, prioritising the list is recommended (Craig et al., 2017). By establishing which barriers and facilitators are considered to have the most important influence but difficult to address in practice, it should help highlight the areas of where the main focus of cessation support interventions should go. This study therefore aimed to develop consensus on the pre-identified barriers and facilitators (Campbell et al., 2018; Flemming et al., 2015), amongst practitioners with experience in providing cessation support to pregnant women, on: a) how influential these barriers and facilitators can be on women's smoking behaviour, and b) how easy or difficult it might be for practitioners to help the women overcome barriers or enhance facilitators. Additionally, using practitioners' suggestions, the study aimed to identify if there are other BCTs that could help address the barriers and facilitators and help pregnant women quit smoking. It also aimed to develop consensus on how appropriate these might be in practice.

## 2 Materials and methods

Ethical approval was granted by the East of Scotland Research Ethics Service REC 1, this covered recruitment from England, UK.

### 2.1 Procedure

The Delphi method is defined as an iterative method in which the range of responses or opinions on the concept in question is reduced with the objective of reaching expert consensus (Helmer-Hirschberg, 1967). This particular method was chosen for this study as, compared with face-to face meetings, it offers a high level of anonymity thereby attracting more honest responses (Keeney, Hasson, & McKenna, 2001), and can cover a large geographical area (Keeney et al., 2001). It has also been successfully used to inform both service provision (Fisher, Walker, Golton, & Jenkinson, 2013) and behavioural change intervention design (Siddiqui et al., 2016). The modified Delphi, a commonly used version, refers to when the material for the first round is mainly derived from relevant data gathered from other resources, prior the Delphi survey commencing (Hsu & Sandford, 2007; Hsu & Sandford, 2010; Kerlinger, 1973).

#### 2.1.1 Survey design and content

Bristol Online Surveys (BOS) software was used to conduct the survey, which entailed sending three different questionnaires to the same respondents in sequential rounds.

##### 2.1.1.1 Round one

In section one of the questionnaire, participants were asked to rate all 34 pre-identified barriers and facilitators (23 barriers, 11 facilitators) (Campbell et al., 2018; Flemming et al., 2015) (listed in Supplement 1) in terms of the importance of influence they had on pregnant women's smoking behaviour. Ratings used a 5-point Likert scale: extremely important (5), very important (4), moderately important (3), slightly important (2), not important (1). In section two, participants were asked to rate the same 34 Barriers and facilitators for ease/difficulty to address in practice on a 5-point Likert scale: very easy (5), easy (4), neither easy nor difficult (3), difficult (2), very difficult (1). In section three, participants were asked for suggestions on ways to address each of the barriers and facilitators in practice. This was done by presenting the full list of barriers and facilitators with a text box next to each one in which respondents were invited to provide open ended responses. To prevent fatigue, they were instructed that there was no upper limit on how many they were expected to provide comments on but encouragement was given to focus on those they thought were the most important and also the most difficult to manage.

##### 2.1.1.2 Round two

For sections one and two, barriers and facilitators that had not reached consensus were re-presented alongside the results from the Round one consensus-rating exercise, and respondents were asked to rate these again as in the first round. In section three, respondent-suggested techniques given in Round one were presented and participants were asked to rate these in terms of appropriateness for use. This was done on a 5-point Likert scale: very appropriate (5), appropriate (4), neither appropriate nor inappropriate (3), inappropriate (2), very inappropriate (1).

##### 2.1.1.3 Round three

Non-consensus reaching barriers and facilitators and respondent-suggested techniques were re-presented with consensus-development results from Round two, respondents were asked to rate these as in previous rounds.

### 2.2 Sampling frame

Practitioners with experience of offering cessation support to pregnant clients, who could commit to completing all three rounds of the survey, were considered eligible. Potential respondents were identified through a register held by the National Centre for Smoking Cessation Training (NCSCT) and from contacts of the Smoking in Pregnancy (SiP) research group at the University of Nottingham (UoN). This included: stop smoking practitioners, stop smoking service leads, midwives, public health representatives, health and wellbeing practitioners, health care assistants and pharmacists. A snowball technique was used in which those contacted initially, either directly by the authors or via the NCSCT, were asked if they could also pass on details of the study to other potentially eligible respondents. It was hoped that the actual sample size would be approximately 50 as, although there is no definitive sample size for a modified Delphi survey, it has been suggested that a larger sample might adversely affect the process as managing responses and response rates can become challenging (Hsu & Sandford, 2007).

### 2.3 Survey distribution

Potential respondents were emailed invitations. They were informed that their involvement was voluntary and responses would be kept confidential and anonymised in any reports. If they agreed to participate, they were emailed a link to the electronic questionnaire with hard copies sent on request for each individual round. Consent was obtained prior to access of any questions. Prior to being circulated, questionnaires were piloted with six researchers from the SiP research group, UoN. In the attempt to minimise loss to follow up, respondents who agreed to take part were asked to provide details of any dates they would be unavailable while the survey was due to run. This influenced the length of time each round remained open to responses for. Approximately one to two weeks before each round was due to start, all eligible respondents were emailed to inform them of the date they would be sent the questionnaire. Between the date of receiving the questionnaire and the date each round was due to close, a maximum of two email reminders were sent to those who were yet to complete it. In compliance with ethical approval, following these emails, if a respondent did not complete a round before it had officially closed, there was no further correspondence unless it was initiated, and entirely voluntary, on the part of the respondent.

### 2.4 Analysis

#### 2.4.1 Delphi consensus

Consensus was considered to be reached on how influential a barrier or facilitator was if ≥70% participants rated it as being either extremely important/very important; or moderately/slightly important; or not important. Similarly, consensus about the ease or difficulty with which practitioners could address individual barriers and facilitators was considered to be reached if ≥70% respondents rated it as being very easy/easy to address; neither easy nor difficult; or very difficult/difficult to address. For the respondent-suggested techniques, consensus was considered to be reached if ≥70% participants rated a technique very inappropriate/inappropriate; neither appropriate nor inappropriate; or very appropriate/appropriate. Total scores, means and standard deviations (SDs) were calculated for all consensus-reaching barriers and facilitators and respondent-suggested techniques in order to ascertain the order of ranking as it was possible that some could have the same percentage of respondent consensus. This follows methods used in previous modified Delphi surveys (Fisher et al., 2013; Gallagher, Bradshaw, & Nattress, 1996; Hagen et al., 2008; Korpershoek, Bruins Slot, Effing, Schuurmans, & Trappenburg, 2017). SPSS v24 was used for analysis carried out on all quantitative data.

#### 2.4.2 Thematic analysis of respondent-suggested techniques and BCT coding

All techniques suggested by the respondents for each of the barriers or facilitators were analysed thematically. This involved highlighting clusters in the data and identifying emerging themes, following methods as described by Braun and Clarke (Braun & Clarke, 2006). Quotes were then extracted that were most representative of the themes identified from all the barriers and facilitators, this was done by three researchers (LF, KAC & TC-H). Additional discussion with two researchers (LF & KAC) resulted in a refinement of this list of quotes to remove any repetition. This list was then presented to three other members of the research team (TC, MU & SC) who gave further suggestions to ensure each quote was distinct from one another. The finalised list was presented to respondents to rate in the questionnaire. For the results to be more meaningful to other researchers and intervention designers, each quote was coded into BCT components. This was done using two existing BCT taxonomies: the behaviour change technique taxonomy version one (BCTTv1) (Michie, Richardson, Johnston, et al., 2013), which is generic to all behaviours and a smoking taxonomy (Michie, Churchill, & West, 2011). As the BCTTv1 (Michie et al., 2013) is the newest, most comprehensive taxonomy (Michie et al., 2013), this was referred to in the first instance. However, as the authors identified that there were some BCTs in the smoking specific taxonomy (Michie, Churchill, & West, 2011) that were not covered by the BCTTv1 (Michie et al., 2013), for any quotes that could not be coded using the BCTTv1 (Michie et al., 2013), the smoking taxonomy (Michie, Churchill, & West, 2011) was then referred to. All coders undertook online training in BCT recognition (UCL, 2011) prior to the analysis. Coding was done independently by two researchers (LF, KAC) and results were compared. Inter-rater reliability for the BCT coding per quote was described using Cohen's Kappa statistic (Cohen, 1960), calculated using SPSS v24. Subsequently, any disagreements were resolved through discussion. If agreement could not be reached through discussion, a third researcher (FL) was consulted.

#### 2.4.3 Behaviour change technique coverage of the barriers and facilitators

To establish the extent of BCT coverage, two researchers (LF & KAC) firstly categorised the barriers and facilitators. Each quote from the list of suggested techniques, with the corresponding BCTs, were then individually mapped to the category of barriers and facilitators they were suggested to address.

## 3 Results

### 3.1 Respondents

In total 167 potential respondents expressed an interest in taking part. Five were not eligible as they practiced outside of England and therefore did not meet with the conditions of ethical approval. Twenty-two practitioners contacted the authors after recruitment had been closed. In total, 140 email invites were sent out. Seventy-eight of the 140 agreed to take part. Fifty-five of the 78 completed the first questionnaire (details of response rates to subsequent rounds are given in Sections 3.2.2 and 3.2.3). Forty-three of the 55 confirmed their job titles. This included: 19 stop smoking practitioners/specialists, eight stop smoking service leads/managers, seven midwives (including research midwives, consultant midwives and midwife cessation trainers), three public health nurse/specialists, three health and wellbeing practitioners (including maternal wellbeing practitioners), two health care assistant/support workers and one community pharmacist. They worked across various settings including antenatal clinics, client's homes, pharmacies, health centres, local authority establishments and NHS clinics. Their years of experience in offering cessation support to pregnant women (for those who reported it, n = 35/55) ranged from 1.5 to 30 years (mean = 10.49 years, SD = 5.79). Each round lasted for a duration of 3–4 weeks during which time respondents could actively complete the questionnaire. There was approximately 1.5 months between rounds which allowed for the analysis to be performed and planned leave due to be taken by the respondents as the survey predominately ran over the summer months.

### 3.2 Delphi consensus

Full results from the Delphi consensus, as described below, are given in Table 1, Table 2, Table 3. Table 1, Table 2 also display the category label that each barrier and facilitator were assigned to, as reported in Section 3.3.

**Table 1:** Barriers and facilitators that gained consensus of importance of influence, in order of ranking.

Barrier or facilitator	Extremely/very important	Total sum of ratings (mean)	Barriers and facilitators category
*Round one*			
Supportive partners (F)	96.4%	256 (4.65)	Influence of significant others
Support and encouragement from family (F)	96.4%	249 (4.53)	Influence of significant others
Having both internal (e.g. for own or baby's health) and external motivation to quit (e.g. for approval of family) (F)	89.1%	245 (4.54)	Motivation & self-efficacy
Meaningful, consistent and personal information about cessation intervention can improve women's engagement (F)	89.1%	247 (4.49)	Aspects of cessation support
Positive relationships with health professional based on trust and mutual respect (F)	89.1%	241 (4.38)	Aspects of cessation support
Lack of support from partners to quit (B)	89.1%	237 (4.31)	Influence of significant others
Lack of support from family to quit (B)	87.3%	229 (4.16)	Influence of significant others
Smoking can help women cope, e.g. with everyday stress (B)	85.5%	219 (3.98)	Stress & general mental well-being
Partners' continued smoking (B)	83.6%	235 (4.27)	Influence of significant others
Poor understanding of risks related to smoking in pregnancy (B)	81.9%	232 (4.13)	Understanding of risks, addiction & withdrawal symptoms
Women want to protect their unborn baby from the harm of smoking (F)	80%	230 (4.18)	Motivation & self-efficacy
Previous experience of quitting can affect current motivation to quit (B)	80%	222 (4.04)	Motivation & self-efficacy
Women don't necessarily see quitting smoking as a priority in their complex lives (B)	76.4%	230 (4.18)	Motivation & self-efficacy
Women lack self-belief in their ability to stop smoking and stay stopped (B)	74.5%	218 (3.96)	Motivation & self-efficacy

*Round two*			
Women underestimate their level of addiction (B)	83.7%	215 (4.39)	Understanding of risks, addiction & withdrawal symptoms
Accurate assessment of the level of tobacco dependence is needed for more appropriate provision of NRT and/or e-cigs (B)	83.7%	212 (4.33)	Aspects of cessation support
Smoking gives women pleasure or brief time out (B)	81.6%	194 (3.96)	Stress & general mental well-being
Women's lack of understanding of how to correctly use NRT (B)	79.2%	197 (4.10)	Aspects of cessation support
Women want to bring up children in smoke-free environment (F)	79.2%	196 (4.08)	Motivation & self-efficacy
Feeling that others disapprove of smoking in pregnancy can make women hide their smoking (B)	75.5%	158 (3.22)	Social norms
Smoking is integral to women's lives and culture (B)	72.9%	188 (3.91)	Social norms
Quitting can make women feel left out if their partner/friends continue to smoke (B)	71.4%	180 (3.81)	Influence of significant others

*Round three*			
Smoking is a social norm, an acceptable behaviour in the women's close social network (B)	88.1%	183 (4.36)	Social norms
Understanding that it is desirable to quit smoking in pregnancy (F)	85.7%	180 (4.29)	Understanding of risks, addiction & withdrawal symptoms
Belief that the stress of quitting will be worse for the baby than continuing to smoke (B)	78.6%	160 (3.81)	Understanding of risks, addiction & withdrawal symptoms
Women's lack of understanding of issues of safety around using NRT in pregnancy (B)	73.8%	168 (4.00)	Aspects of cessation support

(B) denotes barrier, (F) denotes facilitator.

**Table 2:** Barriers and facilitators that gained consensus on ease or difficulty to address in practice, in order of ranking.

Barrier or facilitator	Very easy/easy	Total sum of ratings (mean)	Barriers and facilitators category
*Round one*			
Women's lack of understanding of how to correctly use NRT (B)	87%	217 (3.95)	Aspects of cessation support
Women's lack of understanding of issues of safety around using NRT in pregnancy (B)	85.5%	233 (4.24)	Aspects of cessation support
Accurate assessment of the level of tobacco dependence is needed for more appropriate provision of NRT and/or e-cigs (B)	81.8%	230 (4.18)	Aspects of cessation support
Women want to bring up children in smoke-free environment (F)	80%	220 (4.00)	Motivation & self-efficacy
Women want to protect their unborn baby from the harm of smoking (F)	76.4%	217 (3.95)	Motivation & self-efficacy
Meaningful, consistent and personal information about cessation intervention can improve women's engagement (F)	74.5%	214 (3.89)	Aspects of cessation support

*Round two*			
Positive relationships with health professional based on trust and mutual respect (F)	85.7%	196 (4.00)	Aspects of cessation support
Understanding that it is desirable to quit smoking in pregnancy (F)	85.7%	194 (3.96)	Understanding of risks, addiction & withdrawal symptoms
Women underestimate their level of addiction (B)	77.6%	192 (3.92)	Understanding of risks, addiction & withdrawal symptoms
Poor understanding of risks related to smoking in pregnancy (B)	77.6%	189 (3.86)	Understanding of risks, addiction & withdrawal symptoms
Belief that the stress of quitting will be worse for the baby than continuing to smoke (B)	73.5%	186 (3.80)	Understanding of risks, addiction & withdrawal symptoms
Smoking can help ease boredom (B)	73.5%	184 (3.76)	Stress & general well-being
Fear that quitting smoking could lead to excessive weight gain (B)	73.5%	181 (3.69)	Motivation & self-efficacy
Women underestimate the risks of smoking in pregnancy or don't believe they apply to them (B)	72.9%	173 (3.60)	Understanding of risks, addiction & withdrawal symptoms
Having both internal (e.g. for own or baby's health) and external motivation to quit (e.g. for approval of family) (F)	71.4%	185 (3.78)	Motivation & self-efficacy

*Round three*			
Support and encouragement from family (F)	82.9%	164 (4.00)	Influence of significant others
Supportive partners (F)	78.6%	167 (3.98)	Influence of significant others
Smoking gives women pleasure or brief time out (B)	76.2%	160 (3.81)	Stress & general mental well-being
	Very difficult/difficult	Total sum of ratings (mean)	
Lack of support from partners to quit (B)	71.4%	103 (2.45)	Influence of significant others

(B) denotes barrier, (F) denotes facilitator.

**Table 3:** Behaviour change techniques (BCTs) that gained consensus on appropriateness for use in practice, in order of ranking.

Suggested technique	Very appropriate/appropriate	Total sum of ratings (mean)	Related barriers and facilitators category
*Round two*			
In counselling sessions, provide women with non-judgemental, understanding and consistent support with the same advisor, whenever possible	100%	242 (4.94)	Aspects of cessation support
Assist women on choosing NRT that is right for them, ensure the correct dosage is prescribed/advised upon and provide clear instructions on how and when to use it	100%	239 (4.88)	Aspects of cessation support
Advise on how to use NRT products properly, explaining how these work and emphasise that they are safer than smoking during pregnancy	100%	238 (4.86)	Aspects of cessation support
Provide support early in pregnancy	100%	234 (4.78)	Aspects of cessation support
Assess and discuss cigarette dependence at the first appointment and tailor support accordingly	100%	233 (4.76)	Understanding of risks, addiction & withdrawal symptoms
Reinforce their ideas about wanting to bring up children in a smoke-free environment as being valid	100%	231 (4.71)	Motivation & self-efficacy
Discuss the risks of smoking and benefits of quitting during pregnancy	100%	231 (4.71)	Understanding of risks, addiction & withdrawal symptoms
Prompt the women to make plans to eliminate/avoid triggers to smoke	100%	231 (4.71)	Motivation & self-efficacy
Explain the difference between everyday stress and withdrawal symptoms and how NRT can ease these symptoms	100%	226 (4.61)	Understanding of risks, addiction & withdrawal symptoms
Highlight that experiences from past quit attempts can be turned into positive lessons for this one	100%	226 (4.61)	Motivation & self-efficacy
Identify women's feelings towards and possible impact of partners' continued smoking, encourage them to produce practical solutions regarding this	98%	221 (4.51)	Influence of significant others
Dedicate time in a session to ask questions and listen to women's views, summarise these views back to them	98%	231 (4.71)	Aspects of cessation support
Ensure that women and partners/family members are aware of the dangers of second hand smoke	98%	230 (4.69)	Influence of significant others
Boost their self confidence in being able to quit by giving praise and positive reinforcement	98%	230 (4.69)	Motivation & self-efficacy
Explore and help women find ways to manage negative feelings, such as boredom or stress	98%	229 (4.67)	Stress & general well-being
Help the women to feel confident in being able to experience time out or relieve boredom without a cigarette	98%	227 (4.63)	Stress & general well-being
Establish the stressors in women's lives and explore ways they can manage them	98%	226 (4.61)	Stress & general well-being
Assess women's knowledge and understanding of the risks and tailor information given accordingly	97.9%	220 (4.58)	Understanding of risks, addiction & withdrawal symptoms
Praise women for seeking help	95.9%	227 (4.63)	Aspects of cessation support
Explain that incorrect use of NRT, especially inadequate dosage, can lead to an unsuccessful quit attempt	95.9%	226 (4.61)	Aspects of cessation support
Assess women's levels of motivation to quit and establish ways to build on this	95.9%	225 (4.59)	Motivation & self-efficacy
Advise and support partners/family members to help establish smoke free home by smoking outside	95.9%	224 (4.57)	Influence of significant others
Encourage women to discuss issues surrounding mental well-being and help them to develop coping strategies around this; explain that quitting can lead to making such issues better	95.9%	220 (4.53)	Stress & general mental well-being
Assist women to plan alternative ways to reward herself for not smoking	95.9%	220 (4.53)	Motivation & self-efficacy
Provide support and guidance to help women find the best ways to talk to their family or friends and gain their support with a quit attempt	95.9%	219 (4.47)	Influence of significant others
Explain how smoking can affect mood	95.9%	218 (4.45)	Stress & general well-being
Explore the possible reasons for relapse and plan together to prevent this	95.5%	229 (4.67)	Motivation & self-efficacy
Encourage women to find alternatives to smoking when they are with partners, family members or friends who smoke	93.9%	228 (4.65)	Influence of significant others
Give praise to women who say they want to protect their unborn baby from the harm of smoking	93.9%	227 (4.63)	Motivation & self-efficacy
Be available and flexible for the women that you are providing cessation support to	93.9%	227 (4.63)	Aspects of cessation support
Encourage women's decisions to protect their babies	93.9%	220 (4.53)	Motivation & self-efficacy
Explain the possibility and nature of withdrawal symptoms and give ideas of how to manage them	93.9%	220 (4.53)	Understanding of risks, addiction & withdrawal symptoms
Offer routine CO screening at every counselling session and reinforce treatment based on the results. Highlight improvements in the results	91.8%	233 (4.76)	Aspects of cessation support
Advise partners/family members to smoke outside or vape when with her if they do not want to quit	91.8%	221 (4.51)	Influence of significant others
Assess the factors in women's lives that affect their ability to quit and offer practical advice to make quitting more achievable	91.8%	218 (4.45)	Motivation & self-efficacy
Ask the women to think about what they might gain from being a long term non-smoker	91.8%	215 (4.39)	Motivation & self-efficacy
Involve partners/family members in the treatment process; encourage them to quit with the women	89.9%	220 (4.53)	Influence of significant others
Explore with women why smoking is important to them and why it would be difficult for them to stop	89.8%	219 (4.47)	Motivation & self-efficacy
Explain to women that they will metabolise nicotine faster during pregnancy, how that will make them feel, and why support and NRT are important to help with this	89.6%	217 (4.52)	Aspects of cessation support
Explain to women that although smoking has become part of her life, once they have stopped for a while it will become less normal and they will feel differently about cigarettes	87.8%	205 (4.18)	Motivation & self-efficacy
Ensure women have a good understanding about the nature of addiction	87.5%	213 (4.44)	Understanding of risks, addiction & withdrawal symptoms
Assess the partner's/family members knowledge and understanding of the risks and tailor information given accordingly	85.7%	211 (4.31)	Influence of significant others
Explain that appetite can be altered when quitting and advise on exercise and healthy food choices	85.7%	199 (4.06)	Motivation & self-efficacy
Write smoking cessation notes/advice in handheld or other maternity notes to ensure continuity of care	85.4%	216 (4.50)	Aspects of cessation support
Reassure women that it can take a few attempts to quit and they can be successful this time with support and NRT	83.7%	212 (4.33)	Understanding of risks, addiction & withdrawal symptoms
Inform women that enduring the stress of quitting will be better for the baby than continuing to smoke	75.5%	199 (4.06)	Understanding of risks, addiction & withdrawal symptoms
Build on any sense of guilt, turn it into a positive reason for wanting to quit	75.5%	198 (4.04)	Stress & general well-being
Suggest that the women take up alternative activities which they could do alone or with a social group	71.4%	186 (3.78)	Aspects of cessation support
Discuss and provide support on how to control unhealthy weight gain when quitting smoking	71.3%	191 (3.90)	Motivation & self-efficacy

*Round three*			
Explain the financial benefits of quitting	88.4%	221 (4.51)	Motivation & self-efficacy

#### 3.2.1 Round one

Fourteen of the 34 barriers and facilitators (eight barriers, six facilitators) reached consensus as being extremely/very important in influencing pregnant women's smoking behaviour. These mainly included barriers and facilitators relating to the influence of others in the women's close social network and the women's motivation towards quitting. Those relating to the influence of others were predominately in connection with whether they were supportive (facilitators) or not (barriers). The barriers and facilitators relating to the women's motivation involved both the source of motivation, such as wanting to protect the baby from harm, as well as factors that could impact on it, for example not seeing it as a priority within the complexity of their lives (see Table 1). Six barriers and facilitators (three barriers, three facilitators) reached consensus as being very easy/easy to address in practice. These consisted of barriers relating to women's lack of understanding of how to correctly use nicotine replacement therapy (NRT) and concerns surrounding the safety of using it during pregnancy; and facilitators relating to the women's motivation such as wanting to protect the baby (see Table 2). None reached consensus as being difficult to address.

#### 3.2.2 Round two

Forty-nine of the 55 respondents, who completed Round One, completed this round (89% response rate). Of the 20 barriers and facilitators that had not reached consensus on importance of influence in the previous round, eight (seven barriers, one facilitator) reached consensus as being extremely/very influential. These barriers ranged from women's lack of understanding surrounding their addiction, to the pleasure smoking gives and smoking being integral to the women's lives and culture. The facilitator was the women's want to bring up children in a smoke free environment (see Table 1). A further nine (six barriers, three facilitators) reached consensus as being very easy/easy to address in practice. This mainly included barriers relating to the women's understanding of the risks such as thinking the stress of quitting would be worse for the baby than continued smoking or their underestimation of the risks they are exposed to (see Table 2). None reached consensus as being difficult to address. 54 respondent-suggested techniques were generated in Round one (see Section 3.3), 49 reached consensus as being very appropriate/appropriate. Of the 49, ten reached 100% level of consensus. These ranged from: providing support early in pregnancy, providing correct advice on the use of NRT, highlighting both the risks of continued smoking and the positive aspects surrounding quitting and reinforcing existing, motivating beliefs (see Table 3).

#### 3.2.3 Round three

Forty-four of the 49 participants, who completed the previous rounds, completed this round (90% response rate). Of the 12 barriers and facilitators that had not reached consensus in the previous rounds, four (three barriers, one facilitator) reached consensus as being extremely/very important. These barriers mainly related to smoking being an acceptable social norm and the women's lack of understanding on correct use of NRT and lack of knowledge that it is desirable to quit. On the other hand, the facilitator was that women are aware that quitting would be beneficial (see Table 1) In addition to the 15 barriers and facilitators that had already gained consensus as being easy to address in practice, a further three (one barrier, two facilitators) reached consensus as being very easy/easy to address in practice. The barrier was that smoking could give women pleasure of brief ‘time out.’ The facilitators related to having support and encouragement from the family and partners (see Table 2) One barrier: lack of support from partner to quit, reached consensus as being very difficult/difficult to address (see Table 2). One of the five respondent-suggested techniques: explain the financial benefits of quitting, that did not reach consensus in the previous round reached consensus as being very appropriate/appropriate (see Table 3).

Overall, at the end of the Delphi process, there was practitioner consensus that 26 of the 34 barriers and facilitators were extremely/very important in influencing pregnant smokers smoking. Eighteen reached consensus on being very easy/easy and one on being very difficult/difficult to address in practice. Of the 54 generated respondent-suggested techniques, there was consensus that 50 were very appropriate/appropriate for use with pregnant clients.

### 3.3 Thematic analysis of respondent-suggested techniques and BCT coding

As stated in Section 3.2.2, a list of 54 respondent-suggested techniques that could be used to address the barriers and facilitators was produced (full list given in Supplement 2). For the initial BCT coding from these respondent-suggested techniques by LF and KAC, there was total agreement (kappa = 1) (McHugh, 2012) on 45 of the 54, substantial agreement (kappa 0.61–0.80) (McHugh, 2012) on five and no agreement (kappa ≤ 00) (McHugh, 2012) on three. No BCTS were identified from one. Following further discussion between LF and KAC, for the 9 techniques that had not reached total agreement on the initial coding, total agreement was reached on four and an extra BCT was identified from one. For the remaining five, which included the one there was no coding from, total agreement was obtained following consultation with FL. A further eight BCTs were identified, and reached agreement, from eight of the respondent techniques following recommendations from the publication peer-review process. In total, 33 BCTs were coded from the techniques that reached consensus on being appropriate (see Table 4). Results from the BCT coding process are given in Supplement 3.

### 3.4 Behaviour change technique coverage of the barriers and facilitators

Six distinct categories were identified for the barriers and facilitators: ‘Influence of significant others’, ‘Social norms’, ‘Aspects of cessation support’, ‘Understanding of risks, addiction and withdrawal symptoms’, ‘Stress & general mental well-being’ and ‘Motivation & self-efficacy’ (see Table 5). For the four barriers and two facilitators that fell under the category of ‘Influence of significant others’ there were eight consensus-reaching respondent-suggested techniques, all of which reached consensus as being appropriate. These included a total of four BCTs. Within the ‘Social norms’ category there were four barriers and one facilitator and two non-consensus reaching respondent-suggested techniques, containing two BCTs. There were 13 respondent-suggested techniques for the three barriers and two facilitators within the ‘Aspects of cessation support’ category, 12 of which were consensus-reaching. These contained 16 BCTs. For the four barriers and one facilitator in the ‘Understanding of risks, addiction and withdrawal symptoms’ category there were eight consensus reaching respondent-suggested techniques, containing eight BCTs. There were six respondent-suggested consensus-reaching techniques for the four barriers and one facilitator in the ‘Stress & general mental well-being’ category which contained seven BCTs. For the four barriers and four facilitators in the ‘Motivation & self-efficacy’ category, there were 17 respondent-suggested techniques, 16 of which reached consensus. These contained 16 BCTs. Full results of this analysis are also shown in Table 5.

**Table 4:** BCTs coded from suggested techniques that reached consensus on being very appropriate/appropriate.

BCT category, number and label from the BCTTv1 (23)	Number of times coded from the respondent-suggested techniques
Goals and planning	
1.2 Problem solving	9
Feedback and monitoring	
2.6 Biofeedback	1
Social support	
3.1 Social Support (unspecified)	14
Shaping knowledge	
4.1 Instruction on how to perform the behaviour	3
Natural consequences	
5.1 Information about health consequences	7
5.3 Information about social and environmental consequences	2
5.6 Information about emotional consequences	2
Repetition and substitution	
8.2 Behaviour substitution	2
Comparison of outcomes	
9.2 Pros and cons	1
Reward and threat	
10.4 Social reward	6
10.7 Self-incentive	1
Regulation	
11.1 Pharmacological support	6
11.2 Reduce negative emotions	3
Antecedents	
12.2 Restructuring the social environment	2
12.3 Avoidance/reducing exposure to cues for the behaviour	1
Identity	
13.2 Framing/reframing	3
13.5 Identity associated with changed behaviour	2
Self-belief	
15.1 Verbal persuasion about capability	2
Covert learning	
16.2 Imaginary reward	1


BCT category, number and label (smoking specific BCTs (24) that did not map on to the BCTTV1 (23))	Number of times coded
Specific focus on behaviour maximising self-regulatory capacity/skills	
BS13 Advise on methods of weight control	**1**
General aspects of the interaction focusing on general communication	
RC2 Elicit and answer questions	1
RC4 Explain expectations regarding treatment programme	2
RC6 Provide information on withdrawal symptoms	2
RC7 Use reflective listening	1
RC8 Elicit client views	2
RC9 Summarise information/confirm client decisions	1
RC10 Provide reassurance	1
General aspects of the interaction focusing on delivery of the intervention	
RD1 Tailor interactions appropriately	10
RD2 Emphasise choice	1
General aspects of the intervention focusing on information gathering	
RI2 Assess current readiness and ability to quit	2
RI5 Assess nicotine dependence	1
RI7 Assess attitudes to smoking	1
RI9 Explain how tobacco dependence develops	1

**Table 5:** Categorised barriers and facilitators with related suggested techniques and BCTs coded from these suggestions.

Categorised barriers and facilitators	Related respondent-suggested techniques	BCTs coded
*Influence of significant others*		
Partners' continued smoking (B)^d^	Identify women's feelings towards and possible impact of partners' continued smoking, encourage them to produce practical solutions regarding this^a^	1.2 Problem solving 3.1 Social support (unspecified)
Lack of support from partners to quit (B)^b^, ^d^	Ensure that women and partners/family members are aware of the dangers of second hand smoke^a^	5.1 Information about health consequences
Lack of support from family to quit (B)^d^	Provide support and guidance to help women find the best ways to talk to their family or friends and gain their support with a quit attempt^a^	3.1 Social support (unspecified) RD1 Tailor interactions appropriately
Quitting can make women feel left out if their partner/friends continue to smoke (B)^d^	Encourage women to find alternatives to smoking when they are with partners, family members or friends who smoke^a^	1.2 Problem solving 8.2 Behavioural substitution
Supportive partners (F)^c^, ^d^	Advise partners/family members to smoke outside or vape when with her if they do not want to quit^a^	3.1 Social support (unspecified) 12.2 Restructuring the social environment
Support and encouragement from family (F)^c^, ^d^	Involve partners/family members in the treatment process; encourage them to quit with the women^a^	3.1 Social support (unspecified)
	Assess the partner's/family member's knowledge and understanding of the risks and tailor information given accordingly^a^	RD1 Tailor interactions appropriately
	Advise and support partners/family members to help establish smoke free home by smoking outside^a^	3.1 Social support (unspecified) 12.2 Restructuring of the social environment

*Social norms*		
Smoking is integral to women's lives and culture (B)^d^	Explain that most pregnant women don't smoke; give examples or prevalence rates for from her community where appropriate	6.2 Social comparisons
Feeling that others disapprove of smoking in pregnancy can make women hide their smoking (B)^d^	Explain they are different now as they are pregnant and smoking is not an individual choice any more	13.2 Framing/reframing
Feeling that others disapprove of smoking in pregnancy can lead to quitting smoking (F)^d^		
Smoking is a social norm, an acceptable behaviour in the women's close social network (B)^d^		
Quitting is just for pregnancy; women and their social circle expect that she will go back to smoking after birth (B)^d^		

*Aspects of cessation support*		
Positive relationships with health professional based on trust and mutual respect (F)^c^, ^d^	Praise women for seeking help^a^	3.1 Social support (unspecified) 10.4 Social reward
Accurate assessment of the level of tobacco dependence is needed for more appropriate provision of NRT and/or e-cigs (B)^c^	Explain to women that they will metabolise nicotine faster during pregnancy, how that will make them feel, and why support and NRT are important to help with this^a^	5.1 Information about health consequences 11.1 Pharmacological support RC4 Explain expectations regarding treatment programme
Meaningful, consistent and personal information about cessation intervention can improve women's engagement (F)^c^, ^d^	Advise on how to use NRT products properly, explaining how these work and emphasise that they are safer than smoking during pregnancy^a^	4.1 Instruction on how to perform the behaviour 5.1 Information about health consequences 11.1 Pharmacological support
Women's lack of understanding of how to correctly use NRT (B)^c^	Assist women on choosing NRT that is right for them, ensure the correct dosage is prescribed/advised upon and provide clear instructions on how and when to use it^a^	4.1 Instruction on how to perform the behaviour 11.1 Pharmacological support RD1 Tailor interactions appropriately RD2 Emphasise choice
Women's lack of understanding of issues of safety around using NRT in pregnancy (B)^c^, ^d^	Explain that incorrect use of NRT, especially inadequate dosage, can lead to an unsuccessful quit attempt^a^	11.1 Pharmacological support RC4 Explain expectations regarding treatment programme
	Provide support early in pregnancy^a^	3.1 Social support (unspecified)
	If relevant/possible advise women to attend a social support group which offers cessation support as well as advice on other healthy habits during pregnancy	3.1 Social support (unspecified)
	Suggest that the women take up alternative activities which she could do alone or with a social group^a^	3.1 Social support (unspecified) 8.2 Behaviour substitution
	Offer routine CO screening at every counselling session and reinforce treatment based on the results. Highlight improvements in the results^a^	2.6 Biofeedback RD1 Tailor interactions appropriately
	Dedicate time in a session to ask questions and listen to women's views, summarise these views back to them^a^	RC2 Elicit and answer questions RC7 Use reflective listening RC8 Elicit client views RC9 Summarise information/confirm client decisions
	Write smoking cessation notes/advice in handheld or other maternity notes to ensure continuity of care^a^	4.1 Instruction on how to perform the behaviour 5,3 Information about social and environmental consequences
	Be available and flexible for the women that you are providing cessation support to^a^	3.1 Social support (unspecified)
	In counselling sessions, provide women with non-judgemental, understanding and consistent support with the same advisor, whenever possible^a^	3.1 Social support (unspecified)

*Understanding of risks, addiction and withdrawal symptoms*		
Women underestimate the risks of smoking in pregnancy or don't believe they apply to them (B)^c^	Discuss the risks of smoking and benefits of quitting during pregnancy^a^	5.1 Information about health consequences
Poor understanding of risks related to smoking in pregnancy (B)^c^, ^d^	Explain the possibility and nature of withdrawal symptoms and give ideas of how to manage them^a^	1.2 Problem solving RC6 Provide information on withdrawal symptoms
Belief that the stress of quitting will be worse for the baby than continuing to smoke (B)^c^, ^d^	Inform women that enduring the stress of quitting will be better for the baby than continuing to smoke^a^	5.1 Information about health consequences, 5.6 Information about emotional consequences
Understanding that it is desirable to quit smoking in pregnancy (F)^c^, ^d^	Explain the difference between everyday stress and withdrawal symptoms and how NRT can ease these symptoms^a^	11.1 Pharmacological support RC6 provide information on withdrawal symptoms
Women underestimate their level of addiction (B)^c^	Reassure women that it can take a few attempts to quit and they can be successful this time with support and NRT^a^	11.1 Pharmacological support RC10 Provide reassurance
	Assess women's knowledge and understanding of the risks and tailor information given accordingly^a^	RD1 Tailor interactions appropriately
	Ensure women have a good understanding about the nature of addiction^a^	RI9 Explain how tobacco dependence develops
	Assess and discuss cigarette dependence at the first appointment and tailor support accordingly^a^	RD1 Tailor interactions appropriately RI5 Assess nicotine dependence

*Stress & general mental well-being*		
Smoking can help women cope, e.g. with everyday stress (B)^d^	Explore and help women find ways to manage negative feelings, such as boredom or stress^a^	1.2 Problem solving 11.2 Reduce negative emotions
Smoking gives women pleasure or brief time out (B)^c^	Explain how smoking can affect mood^a^	5.6 Information about emotional consequences
Smoking can help ease boredom (B)^c^	Help the women to feel confident in being able to experience time out or relieve boredom without a cigarette^a^	8.2 Behaviour substitution 11.2 Reduce negative emotions
Fragile mental well-being could be made worse by attempting to stop (B)	Encourage women to discuss issues surrounding mental well-being and help them to develop coping strategies around this; explain that quitting can lead to making such issues better^a^	1.2 Problem solving 5.1 Information about health consequences RD1 Tailor interactions appropriately
Sense of guilt could facilitate attempts to quit smoking (F)	Build on any sense of guilt, turn it into a positive reason for wanting to quit^a^	13.2 Framing/reframing
	Establish the stressors in women's lives and explore ways they can manage them^a^	1.2 Problem solving 11.2 Reduce negative emotions RD1 Tailor interactions appropriately

*Motivation & self-efficacy*		
Fear that quitting smoking could lead to excessive weight gain (B)^c^	Discuss and provide support on how to control unhealthy weight gain when quitting smoking^a^	3.1 Social support (unspecified) BS13 Advise on methods of weight control
Being a smoking mother is seen as a negative thing (e.g. “good mothers” don't smoke) (F)	Explain that appetite can be altered when quitting and advise on exercise and healthy food choices^a^	5.1 Information about health consequences
Women want to protect their unborn baby from the harm of smoking (F)^c^, ^d^	Encourage women's decisions to protect their babies^a^	3.1 Social support (unspecified) 10.4 Social reward
Women want to bring up children in smoke-free environment (F)^c^, ^d^	Give praise to women who say they want to protect their unborn baby from the harm of smoking^a^	3.1 Social support (unspecified) 10.4 Social reward
Women don't necessarily see quitting smoking as a priority in their complex lives (B)^d^	Reinforce their ideas about wanting to bring up children in a smoke-free environment as being valid^a^	10.4 Social reward
Previous experience of quitting can affect current motivation to quit (B)^d^	Assist women to plan alternative ways to reward herself for not smoking^a^	10.7 Self-incentive
Having both internal (e.g. for own or baby's health) and external motivation to quit (e.g. for approval of family) (F)^c^, ^d^	Assess women's levels of motivation to quit and establish ways to build on this^a^	RD1 Tailor interactions appropriately RI2 Assess current readiness and ability to quit
Women lack self-belief in their ability to stop smoking and stay stopped (B)^d^	Boost their self confidence in being able to quit by giving praise and positive reinforcement^a^	10.4 Social reward 15.1 Verbal persuasion about capability
	Prompt the woman to make plans to eliminate/avoid triggers to smoke^a^	1.2 Problem solving 12.3 Avoidance/reducing exposure to cues for the behaviour
	Explain the financial benefits of quitting^a^	5.3 Information about social and environmental consequences
	Explain to women that although smoking has become part of life, once they have stopped for a while it will become less normal and they will feel differently about cigarettes^a^	13.2 Framing/reframing
	Ask the women to think about what she might gain from being a long term non-smoker^a^	9.2 Pros and cons 13.5 Identity associated with changed behaviour 16.2 Imaginary reward
	Ask women to imagine how they would feel about a child or a baby smoking	5.5 Anticipated regret
	Highlight that experiences from past quit attempts can be turned into positive lessons for this one^a^	15.1 Verbal persuasion about capability
	Assess the factors in women's lives that affect their ability to quit and offer practical advice to make quitting more achievable^a^	1.2 Problem solving RD1 Tailor interactions appropriately
	Explore with women why smoking is important to them and why it would be difficult for them to stop^a^	RC8 Elicit client views RI7 Assess attitudes to smoking
	Explore the possible reasons for relapse and plan together to prevent this^a^	1.2 Problem solving

(B) denotes barrier, (F) denotes facilitator.

a A respondent-suggested technique reached consensus as being appropriate for use.

b A B or F that reached consensus as being difficult to address.

c A B or F that reached consensus as being easy to address.

d A B or F that reached consensus as being influential.

## 4 Discussion

This study prioritised pre-identified barriers and facilitators, from the perspective of practitioners, in terms of the importance of influence they can have on pregnant women's smoking behaviour and how easy or difficult they could be to manage in practice. It also generated suggestions on how to address them, from which 33 BCTs were identified. The BCTs coded most frequently from the respondent-suggested techniques were ‘Social support (unspecified)’ (Michie et al., 2013), ‘Tailor interactions appropriately’ (Michie, Churchill, & West, 2011) and ‘Problem solving’ (Michie et al., 2013). Having a ‘supportive partner’ was rated as the most influential facilitator whereas ‘lack of support from partner’ was the only barrier that reached consensus as being difficult to address. The barriers and facilitators that fell under the category of ‘Social norms’, all of which reached consensus as being influential, lacked coverage of consensus reaching respondent-suggested techniques.

The importance of influence that partners were perceived to have reflects that of existing evidence which shows a relatively consistent association between partner's support, smoking status and the outcome of pregnant women's quit attempts (Riaz, Lewis, Naughton, & Ussher, 2018), with women being more likely to continue smoking during their pregnancy if their partner smokes (Lemola & Grob, 2008). As such, partners' influence would seem a priority to focus on, especially as having an unsupportive partner was the only barrier in this study that reached consensus as being difficult to address. The BCT ‘Social support (unspecified)’ (Michie et al., 2013) was coded often from the respondent-suggested techniques given for this purpose. Including the partner in the treatment process and encouraging them to quit with the women were some examples that reached consensus as being appropriate. When using this technique in practice, it may help practitioners to understand the potential different reasons behind partners' continued smoking; for example they may lack awareness of any risk that they are posing to the unborn baby (Bottorff, Oliffe, Kalaw, Carey, & Mroz, 2006). If the partner is continuously unsupportive, or if the dynamics between the women and partner are conflicting in regards to smoking cessation, other avenues of support could be encouraged (Hemsing, Greaves, O'leary, Chan, & Okoli, 2011). For example, a review highlighted that using non-smoking female buddies was an effective way to encourage cessation (Albrecht, Payne, Stone, & Reynolds, 1998). Also, in work relating to alcohol consumption reduction during pregnancy, it was found to be useful when the women decided who would partner them for the duration of an intervention and specified what type of support they would like that other person to provide them with (Chang et al., 2005). These alternatives may be useful especially as it is known that partners are less likely to receive cessation support with the women (Hemsing et al., 2011) making them less accessible to practitioners. A systematic review also found that supplying intervention materials to the women to pass on to their partners appeared to be ineffective in terms of cessation attempts made by partners and the level of support they provide to the women (Hemsing et al., 2011).

‘Problem solving’ (Michie et al., 2013) was another BCT coded most frequently from the respondent-suggested techniques relating to the influence significant others, in general, could have on the women's smoking habits. This included ensuring that the women and these significant others were aware of the dangers of continued smoking and helping the women find solutions to overcome any negative influence these people may have.

As the barriers that fell under the ‘Social Norms’ category received poor coverage of respondent-suggested techniques, despite all reaching consensus as being influential, it highlights an area that requires more focus in terms of establishing what could be useful in helping women overcome these particular barriers. The importance of influence relating to social norms, in general, has also been highlighted in other work, taken from different perspectives. For example, qualitative work of pregnant smoker's experiences, identified the theme of ‘living in a smoking world’ as being the most important, overarching theme (Murray, Small, & Burrage, 2014). From this theme, it was highlighted that not only could smoking become normalised and perceptions of the risks minimised if the women lived in communities that were highly populated with smokers; if most of their family members smoked, they felt that their smoking status was inherent and as such, not something they had full, or much, autonomy over (Murray et al., 2014). It is therefore possible that the two respondent-suggested techniques relating to barriers in this category, which coded as ‘Social comparison’ and ‘Framing/reframing’ (Michie et al., 2013), did not reach consensus as being appropriate as they included explaining that it is not the norm for women to smoke during pregnancy in her local area. Also, the suggestion of telling women that now they are pregnant, smoking is not an individual choice anymore may have been perceived as being confusing by practitioners who have had any experience of women holding the belief that being a smoker is not something they had much control or choice over in the first instance (Murray et al., 2014).

Facilitators relating to ‘Motivation & self-efficacy’, for example, ‘women want to protect their baby from the harm of smoking’ were also considered particularly influential. This reflects evidence to show that wanting to protect the baby is a significant predictor of women successfully quitting (Ripley-Moffitt et al., 2008; Wakschlag et al., 2003). However, certain barriers that could potentially override this motivation, for example ‘women do not necessarily see quitting smoking as a priority in their complex lives’ were also considered important. Positive behaviour change is only thought to be achievable when the level of motivation to change exceeds the desire to continue engaging in the unwanted behaviour (West & Brown, 2013). In relation to smoking, it has been suggested that more successful cessation attempts can be achieved if this optimal level of motivation remains stable over time (Perski, Herd, Brown, & West, 2018). One particular respondent-generated technique from this study ‘assess women's levels of motivation to quit and establish ways to build on this’ could help to address this, especially if done at each consultation and not just used as a one-off technique. The respondent-suggested technique of providing consistent support would support using this type of approach. Respondent-suggested techniques that involved giving positive reinforcement and praise, coded mainly as ‘Social reward’ (Michie et al., 2013), were also deemed particularly appropriate for barriers and facilitators relating to ‘Motivation & self-efficacy’.

## 5 Future work

In the case for women who lack support from their partner, as mentioned previously, having non-smoking female buddies has been shown to help (Albrecht et al., 1998) as could giving the women the opportunity to choose who supports them and how (Chang et al., 2005). However, the feasibility of these alternatives may need further exploration, especially as ‘smoking is a social norm, an acceptable behaviour in the women's close social network’ was a barrier that reached consensus as having an important influence within this study. It therefore be more difficult to for women who experience this barrier to identify a suitable non-smoking buddy in their close circle of family and friends. Also, to the author's knowledge, the latter of these suggestions has been tested in alcohol reduction studies only (Chang et al., 2005) and not yet for smoking cessation during pregnancy.

Establishing how to effectively address barriers relating to ‘Social norms’ was also identified as a priority for further, more in-depth research. Findings from previous work which shows that women feel that their smoking habits are inherent and as such, they do not feel that they have much autonomy over choices made in regards to smoking (Murray et al., 2014), follows the patterns that people are less likely to make positive health behaviour choices if their perceived internal control over them is low (Cobb-Clark, Kassenboehmer, & Schurer, 2014). Smokers in the general population have been found to respond well, and be more likely to achieve abstinence when exposed to cessation support interventions that are aimed to boost perceived autonomy and develop a sense of self-competency towards achieving the desired behavioural outcome (Williams et al., 2006). This may be worth trialling with pregnant women and is an aspect that is in line with some of the respondent-suggested techniques that related to the barriers and facilitators that fell under the ‘Motivation & self-efficacy’ category. Further qualitative work with stop smoking practitioners would also help to explore why suggestions on how to address barriers relating to ‘Social norms’ were so sparse and if there are any alternative BCTs that could be useful when addressing these in practice.

## 6 Strengths and limitations

The Delphi process relies on the existing knowledge of the respondents and therefore may miss new, novel and other relevant ideas or issues (Black et al., 1999). To minimise this potential limitation, we recruited a wide range of experienced practitioners, based in various settings. As the context in which cessation support interventions are delivered can have a significant impact on intervention outcomes (Peters, de Bruin, & Crutzen, 2015) recruiting in this manner also allowed responses to be gathered that account for many different contextual factors. The high response rate to the survey was also a strength. As recruitment was done from England only however, the findings may not be relevant or appropriate to areas outside the UK. As interventions tend to translate poorly and do not produce the same outcomes across different countries mainly due to cultural differences (De Vries et al., 2003), recruiting from other countries may therefore have been more of a limitation to this study. It is possible that there may be regional differences in England with regards to the barriers and facilitators that women may experience, however the expert group study in which the list of barriers and facilitators was refined and finalised (Campbell et al., 2018), purposefully recruited participants from various regions of the UK which helped ensure that differences of this type could be taken into consideration.

Typically, studies aiming to identify potentially effective BCTs, describe the BCTs using labels from a specific behaviour change technique taxonomy only, which can be susceptible to subjective interpretation (Ogden, 2016). By reporting full descriptions of respondent-suggested techniques alongside the corresponding BCT labels from the relevant BCT taxonomy (Michie, Churchill, & West, 2011; Michie et al., 2013) it not only indicated which BCTs could be useful, but also gave descriptions of how they could be operationalised in practice. The high level of consensus on the appropriateness of the respondent-suggested techniques can also be considered as a strength of the study, however, it is also possible that consensus building on these techniques could have been biased as they were based on the responses given by the practitioners themselves. In order to overcome this we ensured that opposing views in the suggestions were reflected in the final list of quotes, although this may not completely negate this potential risk of response bias.

## 7 Conclusions

The results highlight the important influence that smoking cessation practitioners perceive partners to have on pregnant women's smoking behaviour and that having an unsupportive partner is thought to the most difficult barrier to manage in consultations. Involving partners to engage in, and offer support during a quit attempt was advocated, however if the partner is unsupportive, enlisting support from suitable others may help. Appropriate ways of how to address barriers surrounding ‘Social norms’ were not well established. Giving consistent support and boosting motivation were considered relatively easy to address and beneficial.

The following are the supplementary data related to this article.

Supplement 1 34 pre-identified barriers and facilitators. Supplement 1

Supplement 2 Final List of 54 respondent-suggested techniques. Supplement 2

Supplement 3 Full results of the behaviour change technique coding process from the respondent-suggested techniques. Supplement 3

## Conflict of interest

All authors declare that they have no conflicts of interest.
