# Supplementary material for: Stop smoking practitioner consensus on barriers and facilitators to smoking cessation in pregnancy and how to address these: A modified Delphi survey
Source: Addict Behav Rep. 2019 Jan 29;9:100164. doi: 10.1016/j.abrep.2019.100164 (PMC6543497; doi:10.1016/j.abrep.2019.100164)
Supplement: Supplement 1 — 34 pre-identified barriers and facilitators. [file mmc1.docx]

Supplement 1: 34 pre-identified barriers and facilitators

(B) denotes barrier, (F) denotes facilitator

- Smoking is integral to women's lives and culture (B)
- Smoking can help women cope, e.g. with everyday stress (B)
- Smoking gives women pleasure or brief time out (B)
- Smoking can help ease boredom (B)
- Fragile mental well-being could be made worse by attempting to stop (B)
- Fear that quitting smoking could lead to excessive weight gain (B)
- Feeling that others disapprove of smoking in pregnancy can make women hide their smoking (B)
- Feeling that others disapprove of smoking in pregnancy can lead to quitting smoking (F)
- Sense of guilt could facilitate attempts to quit smoking (F)
- Smoking is a social norm, an acceptable behaviour in the women's close social network (B)
- Partners’ continued smoking (B)
- Lack of support from partners to quit (B)
- Lack of support from family to quit (B)
- Quitting can make women feel left out if their partner/friends continue to smoke (B)
- Supportive partners (F)
- Support and encouragement from family (F)
- Positive relationships with health professional based on trust and mutual respect (F)
- Women underestimate the risks of smoking in pregnancy or don’t believe they apply to them (B)
- Poor understanding of risks related to smoking in pregnancy (B)
- Belief that the stress of quitting will be worse for the baby than continuing to smoke (B)
- Understanding that it is desirable to quit smoking in pregnancy (F)
- Quitting is just for pregnancy; women and their social circle expect that she will go back to smoking after birth (B)
- Being a smoking mother is seen as a negative thing (e.g. "good mothers" don't smoke) (F)
- Women want to protect their unborn baby from the harm of smoking (F)
- Women want to bring up children in smoke-free environment (F)
- Women's lack of understanding of how to correctly use NRT (B)
- Women's lack of understanding of issues of safety around using NRT in pregnancy (B)
- Women underestimate their level of addiction (B)
- Women don’t necessarily see quitting smoking as a priority in their complex lives (B)
- Previous experience of quitting can affect current motivation to quit (B)
- Having both internal (e.g. for own or baby's health) and external motivation to quit (e.g. for approval of family) (F)
- Women lack self-belief in their ability to stop smoking and stay stopped (B)
- Accurate assessment of the level of tobacco dependence is needed for more appropriate provision of NRT and/or e-cigs (B)
- Meaningful, consistent and personal information about cessation intervention can improve women's engagement (F)
