# Supplementary material for: Stop smoking practitioner consensus on barriers and facilitators to smoking cessation in pregnancy and how to address these: A modified Delphi survey
Source: Addict Behav Rep. 2019 Jan 29;9:100164. doi: 10.1016/j.abrep.2019.100164 (PMC6543497; doi:10.1016/j.abrep.2019.100164)
Supplement: Supplement 2 — Final List of 54 respondent-suggested techniques. [file mmc2.docx]

Supplement 2: Final List of 54 respondent-suggested techniques

- Identify women’s feelings towards and possible impact of partners’ continued smoking, encourage them to produce practical solutions regarding this.
- Encourage women to find alternatives to smoking when they are with partners, family members or friends who smoke.
- Ensure that women and partners/family members are aware of the dangers of second hand smoke.
- Involve partners/family members in the treatment process; encourage them to quit with the women.
- Provide support and guidance to help women find the best ways to talk to their family or friends and gain their support with a quit attempt.
- Advise partners/family members to smoke outside or vape when with her if they do not want to quit.
- Advise and support partners/family members to help establish smoke free home by smoking outside.
- Establish the stressors in women’s lives and explore ways they can manage them.
- Explore and help women find ways to manage negative feelings, such as boredom or stress.
- Inform women that enduring the stress of quitting will be better for the baby than continuing to smoke.
- Encourage women to discuss issues surrounding mental well-being and help them to develop coping strategies around this; explain that quitting can lead to making such issues better.
- Encourage women’s decisions to protect their babies.
- Explain they are different now as they are pregnant and smoking is not an individual choice any more.
- Praise women for seeking help.
- Boost their self confidence in being able to quit by giving praise and positive reinforcement.
- Assess women’s levels of motivation to quit and establish ways to build on this.
- Build on any sense of guilt, turn it into a positive reason for wanting to quit.
- Help the women to feel confident in being able to experience time out or relieve boredom without a cigarette.
- Assist women to plan alternative ways to reward herself for not smoking.
- Give praise to women who say they want to protect their unborn baby from the harm of smoking.
- Reinforce their ideas about wanting to bring up children in a smoke-free environment as being valid.
- Discuss the risks of smoking and benefits of quitting during pregnancy.
- Assess women’s knowledge and understanding of the risks and tailor information given accordingly.
- Assess the partner’s/ family member’s knowledge and understanding of the risks and tailor information given accordingly.
- Explain the financial benefits of quitting.
- Explain to women that although smoking has become part of life, once they have stopped for a while it will become less normal and they will feel differently about cigarettes.
- Ask the women to think about what she might gain from being a long term non-smoker.
- Explain how smoking can affect mood.
- Ask women to imagine how they would feel about a child or a baby smoking.
- Ensure women have a good understanding about the nature of addiction.
- Explain to women that they will metabolise nicotine faster during pregnancy, how that will make them feel, and why support and NRT are important to help with this.
- Advise on how to use NRT products properly, explaining how these work and emphasis that they are safer than smoking during pregnancy.
- Assist women on choosing NRT that is right for them, ensure the correct dosage is prescribed/advised upon and provide clear instructions on how and when to use it.
- Explain that incorrect use of NRT, especially inadequate dosage, can lead to an unsuccessful quit attempt.
- Explain the difference between everyday stress and withdrawal symptoms and how NRT can ease these symptoms.
- Reassure women that it can take a few attempts to quit and they can be successful this time with support and NRT.
- Provide support early in pregnancy.
- If relevant / possible advise women to attend a social support group which offers cessation support as well as advice on other healthy habits during pregnancy.
- Discuss and provide support on how to control unhealthy weight gain when quitting smoking.
- Explain that appetite can be altered when quitting and advise on exercise and healthy food choices.
- Explain that most pregnant women don't smoke; give examples or prevalence rates for from her community where appropriate.
- Suggest that the women take up alternative activities which she could do alone or with a social group.
- Explain the possibility and nature of withdrawal symptoms and give ideas of how to manage them.
- Explore the possible reasons for relapse and plan together to prevent this.
- Prompt the woman to make plans to eliminate/avoid triggers to smoke.
- Be available and flexible for the women that you are providing cessation support to.
- In counselling sessions, provide women with non-judgemental, understanding and consistent support with the same **advisor**, whenever possible.
- Assess and discuss cigarette dependence at the first appointment and tailor support accordingly.
- Offer routine CO screening at every counselling session and reinforce treatment based on the results. Highlight improvements in the results.
- Dedicate time in a session to ask questions and listen to women’s views, summarise these views back to them.
- Highlight that experiences from past quit attempts can be turned into positive lessons for this one.
- Write smoking cessation notes/advice in handheld or other maternity notes to ensure continuity of care.
- Assess the factors in women’s lives that affect their ability to quit and offer practical advice to make quitting more achievable.
- Explore with women why smoking is important to them and why it would be difficult for them to stop.
