# Supplementary material for: Stop smoking practitioner consensus on barriers and facilitators to smoking cessation in pregnancy and how to address these: A modified Delphi survey
Source: Addict Behav Rep. 2019 Jan 29;9:100164. doi: 10.1016/j.abrep.2019.100164 (PMC6543497; doi:10.1016/j.abrep.2019.100164)
Supplement: Supplement 3 — Full results of the behaviour change technique coding process from the respondent-suggested techniques. [file mmc3.docx]

Supplement 3: Full results of the behaviour change technique coding process from the respondent-suggested techniques

|  | **Initial BCTs Coded** | |  |
| --- | --- | --- | --- |
| **Suggested techniques** | **LF** | **KAC** | **Final agreement** |
| Identify women’s feelings towards and possible impact of partners’ continued smoking, encourage them to produce practical solutions regarding this | 1.2, 3.1 | 1.2, 3.1 | 1.2, 3.1 |
| Encourage women to find alternatives to smoking when they are with partners, family members or friends who smoke | 1.2, 8.2 | 1.2, 8.2 | 1.2, 8.2 |
| Ensure that women and partners/family members are aware of the dangers of second hand smoke | 5.1 | 5.1 | 5.1 |
| Involve partners/family members in the treatment process; encourage them to quit with the women | 3.1 | 3.1 | 3.1 |
| Provide support and guidance to help women find the best ways to talk to their family or friends and gain their support with a quit attempt | 3.1, RD1 | 3.1 | 3.1, RD1 |
| Advise partners/family members to smoke outside or vape when with her if they do not want to quit | 3.1 | 3.1 | 3.1, 12.2*** |
| Advise and support partners/family members to help establish smoke free home by smoking outside | 3.1 | 3.1 | 3.1, 12.2*** |
| Establish the stressors in women’s lives and explore ways they can manage them | 1.2, RD1 | 1.2, 11.2, RD1 | 1.2, 11.2, RD1 |
| Explore and help women find ways to manage negative feelings, such as boredom or stress | 1.2, 11.2 | 1.2, 11.2 | 1.2, 11.2 |
| Inform women that enduring the stress of quitting will be better for the baby than continuing to smoke | 5.1, 5.6 | 5.1, 5.6 | 5.1, 5.6 |
| Encourage women to discuss issues surrounding mental well-being and help them to develop coping strategies around this; explain that quitting can lead to making such issues better | 1.2, 5.1, RD1 | 1.2, 5.1 | 1.2, 5.1, RD1 |
| Encourage women’s decisions to protect their babies | 10.4 | 10.4 | 10.4, 3.1*** |
| Praise women for seeking help | 10.4 | 10.4 | 10.4, 3.1*** |
| Boost their self confidence in being able to quit by giving praise and positive reinforcement | 10.4, 15.1 | 10.4, 15.1 | 10.4, 15.1 |
| Assess women’s levels of motivation to quit and establish ways to build on this | RD1, RI2 | RD1, RI2 | RD1, RI2 |
| Build on any sense of guilt, turn it into a positive reason for wanting to quit | 13.2 | 13.2 | 13.2 |
| Help the women to feel confident in being able to experience time out or relieve boredom without a cigarette | 1.2, 8.2, 11.2 | 8.2, 11.2 | 8.2, 11.2 |
| Assist women to plan alternative ways to reward herself for not smoking | 10.7 | 10.7 | 10.7 |
| Give praise to women who say they want to protect their unborn baby from the harm of smoking | 10.4 | 10.4 | 10.4, 3.1*** |
| Reinforce their ideas about wanting to bring up children in a smoke-free environment as being valid | 10.4 | 10.4 | 10.4 |
| Discuss the risks of smoking and benefits of quitting during pregnancy | 5.1 | 5.1 | 5.1 |
| Assess women’s knowledge and understanding of the risks and tailor information given accordingly | RD1 | RD1 | RD1 |
| Assess the partner’s/ family member’s knowledge and understanding of the risks and tailor information given accordingly | RD1 | RD1 | RD1 |
| Explain the financial benefits of quitting | No coding | 5.3 | 5.3** |
| Explain to women that although smoking has become part of life, once they have stopped for a while it will become less normal and they will feel differently about cigarettes | 5.6 | 13.2, 13.5 | 13.2** |
| Ask the women to think about what she might gain from being a long term non-smoker | 9.2, 13.5 | 9.2, 13.5, 16.2 | 9.2, 13.5, 16.2 |
| Explain how smoking can affect mood | 5.6 | 5.6 | 5.6 |
| Ensure women have a good understanding about the nature of addiction | No coding | RI9 | RI9** |
| Explain to women that they will metabolise nicotine faster during pregnancy, how that will make them feel, and why support and NRT are important to help with this | 5.1, 11.1, RC4 | 5.1, 11.1, RC4 | 5.1, 11.1, RC4 |
| Advise on how to use NRT products properly, explaining how these work and emphasis that they are safer than smoking during pregnancy | 4.1, 5.1, 11.1 | 4.1, 5.1, 11.1 | 4.1, 5.1, 11.1 |
| Assist women on choosing NRT that is right for them, ensure the correct dosage is prescribed/advised upon and provide clear instructions on how and when to use it | 4.1, 11.1, RD1 | 4.1, 11.1, RD2 | 4.1, 11.1, RD1, RD2 |
| Explain that incorrect use of NRT, especially inadequate dosage, can lead to an unsuccessful quit attempt | 11.1 | 11.1 | 11.1, RC4 |
| Explain the difference between everyday stress and withdrawal symptoms and how NRT can ease these symptoms | 11.1, RC6 | 11.1, RC6 | 11.1, RC6 |
| Reassure women that it can take a few attempts to quit and they can be successful this time with support and NRT | 11.1, RC10 | 11.1, RC10 | 11.1, RC10 |
| Provide support early in pregnancy | 3.1 | 3.1 | 3.1 |
| Discuss and provide support on how to control unhealthy weight gain when quitting smoking | 3.1 | 3.1 | 3.1, BS13*** |
| Explain that appetite can be altered when quitting and advise on exercise and healthy food choices | 5.1 | 5.1 | 5.1 |
| Suggest that the women take up alternative activities which she could do alone or with a social group | 3.1, 8.2 | 3.1, 8.2 | 3.1, 8.2 |
| Explain the possibility and nature of withdrawal symptoms and give ideas of how to manage them | 1.2, RC6 | 1.2, RC6 | 1.2, RC6 |
| Explore the possible reasons for relapse and plan together to prevent this | 1.2 | 1.2 | 1.2 |
| Prompt the woman to make plans to eliminate/avoid triggers to smoke | 1.2, 12.3 | 12.3 | 1.2, 12.3 |
| Be available and flexible for the women that you are providing cessation support to | 3.1 | 3.1 | 3.1 |
| In counselling sessions, provide women with non-judgemental, understanding and consistent support with the same advisor, whenever possible | 3.1 | 3.1 | 3.1 |
| Assess and discuss cigarette dependence at the first appointment and tailor support accordingly | RD1, RI5 | RD1, RI5 | RD1, RI5 |
| Offer routine CO screening at every counselling session and reinforce treatment based on the results. Highlight improvements in the results | 2.6, RD1 | 2.6, RD1 | 2.6, RD1 |
| Dedicate time in a session to ask questions and listen to women’s views, summarise these views back to them | RC2, RC7, RC8, RC9 | RC2, RC7, RC8, RC9 | RC2, RC7, RC8, RC9 |
| Highlight that experiences from past quit attempts can be turned into positive lessons for this one | 15.1 | 15.1 | 15.1 |
| Write smoking cessation notes/advice in handheld or other maternity notes to ensure continuity of care | ? no coding | ? no coding | 4.1, 5.3** |
| Assess the factors in women’s lives that affect their ability to quit and offer practical advice to make quitting more achievable | 1.2, RD1 | 1.2, RD1 | 1.2, RD1, RI2*** |
| Explore with women why smoking is important to them and why it would be difficult for them to stop | ? RC8 | ? RC8 | RC8**, RI7*** |
|  |  |  |  |
| Explain they are different now as they are pregnant and smoking is not an individual choice any more * | 13.2 | 13.2 | 13.2 |
| Ask women to imagine how they would feel about a child or a baby smoking * | ? 5.2 | ? 5.2 | 5.2** |
| If relevant / possible advise women to attend a social support group which offers cessation support as well as advice on other healthy habits during pregnancy * | 3.1 | 3.1 | 3.1 |
| Explain that most pregnant women don't smoke; give examples or prevalence rates for from her community where appropriate * | 6.2 | 6.2 | 6.2 |

** Respondent suggested techniques that did not reach consensus on being appropriate for use in practice*

*? denotes coders were uncertain*

***Final coding agreed on consultation with FL*

****Code added following recommendations from the peer-review process*
